# Young-onset sporadic Creutzfeldt–Jakob disease with atypical phenotypic features: a case report

**DOI:** 10.1186/s13256-019-2089-5

**Published:** 2019-05-29

**Authors:** Durjoy Lahiri, Srimant Pattnaik, Ashwani Bhat, Souvik Dubey, Atanu Biswas, Biman Kanti Roy

**Affiliations:** Department of Neurology, Bangur Institute of Neurosciences, IPGMER and SSKM Hospital, Kolkata, West Bengal 700025 India

**Keywords:** Case report, Creutzfeldt–Jakob disease, Sporadic, Young

## Abstract

**Background:**

Sporadic Creutzfeldt–Jakob disease, with a mean survival of 6 months, is duly considered among the most fatal neurological disorders. Rapidly progressive dementia with multi-axial involvement of the nervous system is the known presentation. Although, the peak age at onset is between sixth and eighth decades, cases of young-onset sporadic Creutzfeldt–Jakob disease have also been reported in the literature. Interestingly, these young-onset cases were reported to have some features distinct from their older age group counterparts, such as slower progression as well as longer duration of illness, dominance of psychiatric manifestations at the onset, and relatively less prevalence of radiological and electroencephalographic abnormalities.

**Case presentation:**

We describe here the case of a 42-year-old Asian woman from India who presented with cerebellar ataxia, pyramidal and extrapyramidal involvement, followed by rapidly progressive dementia along with myoclonus, all within a span of 1 month. Probable infective, metabolic, autoimmune, and paraneoplastic etiologies were ruled out. Magnetic resonance imaging of her brain revealed bilateral caudate nucleus hyperintensity in T_2_/fluid-attenuated inversion recovery sequence. Diffusion-weighted imaging revealed bilateral caudate and putaminal diffusion restriction plus ribbon pattern in bilateral parieto-occipital and insular cortex. Serial electroencephalography revealed diffuse slowing of background activity along with triphasic waves in short periodic interval. Cerebrospinal fluid was tested positive for 14-3-3 protein. Based on these findings, a diagnosis of sporadic Creutzfeldt–Jakob disease was made.

**Conclusion:**

Our patient represents an atypical clinical situation as she is much younger than the usual presentation of Creutzfeldt–Jakob disease and it progressed far too rapidly. Cognitive decline came late in the temporal sequence of clinical events; rather, the onset was dominated by features consistent with cerebellar ataxia and basal ganglia involvement. The presence of magnetic resonance imaging abnormality and electroencephalography changes are other rare findings in young-onset sporadic Creutzfeldt–Jakob disease.

## Background

Prion diseases are a group of neurodegenerative diseases caused by the conversion of the normal form of prion protein (PrPC, prion-related protein, in which C stands for the cellular form of the protein) with a primarily alpha-helical structure into an abnormal form of the prion protein (PrPSc, proteinaceous infectious particle, in which Sc stands for scrapie, the prion disease of sheep and goats), which has a primarily beta-pleated sheet structure. Prion diseases are unusual in medicine in that they can occur by three mechanisms: spontaneous (sporadic), genetic (familial), and acquired (infectious/transmitted). Sporadic forms of prion disease include sporadic Creutzfeldt–Jakob disease (CJD) and the recently identified variably protease-sensitive prionopathy. Sporadic CJD, the most common form of human prion disease, represents a catastrophic form of neurodegeneration. The peak age of onset is between 55 and 75 years and the mean survival is 6 months only [1, 2]. Rapidly progressive dementia usually dominates the clinical presentation in a backdrop of multi-axial involvement manifested by cerebellar ataxia, pyramidal and extrapyramidal involvement, visual dimness and myoclonus. However, cases of young-onset sporadic CJD have also been reported in the literature, although only few in number. These young-onset cases are, to some extent, similar to variant CJD by virtue of the fact that they have a slower course, long duration of illness, and dominant psychiatric manifestations at onset. On the other hand, they also share some of the radiological, biochemical, and electroencephalographic features of sporadic CJD in the older age group. However, magnetic resonance imaging (MRI) and electroencephalography (EEG) abnormalities are far more common in the older group than in the cases of young-onset CJD [3]. We present here a case of young-onset sporadic CJD with some unusual clinical features like too rapid deterioration, relatively late appearance of dementia, and dominance of cerebellar ataxia with extrapyramidal feature at the onset of illness. Furthermore, our case exhibits almost typical MRI and EEG findings of sporadic CJD, besides being positive for 14-3-3 protein cerebrospinal fluid (CSF).

## Case presentation

**Fig. 1:**
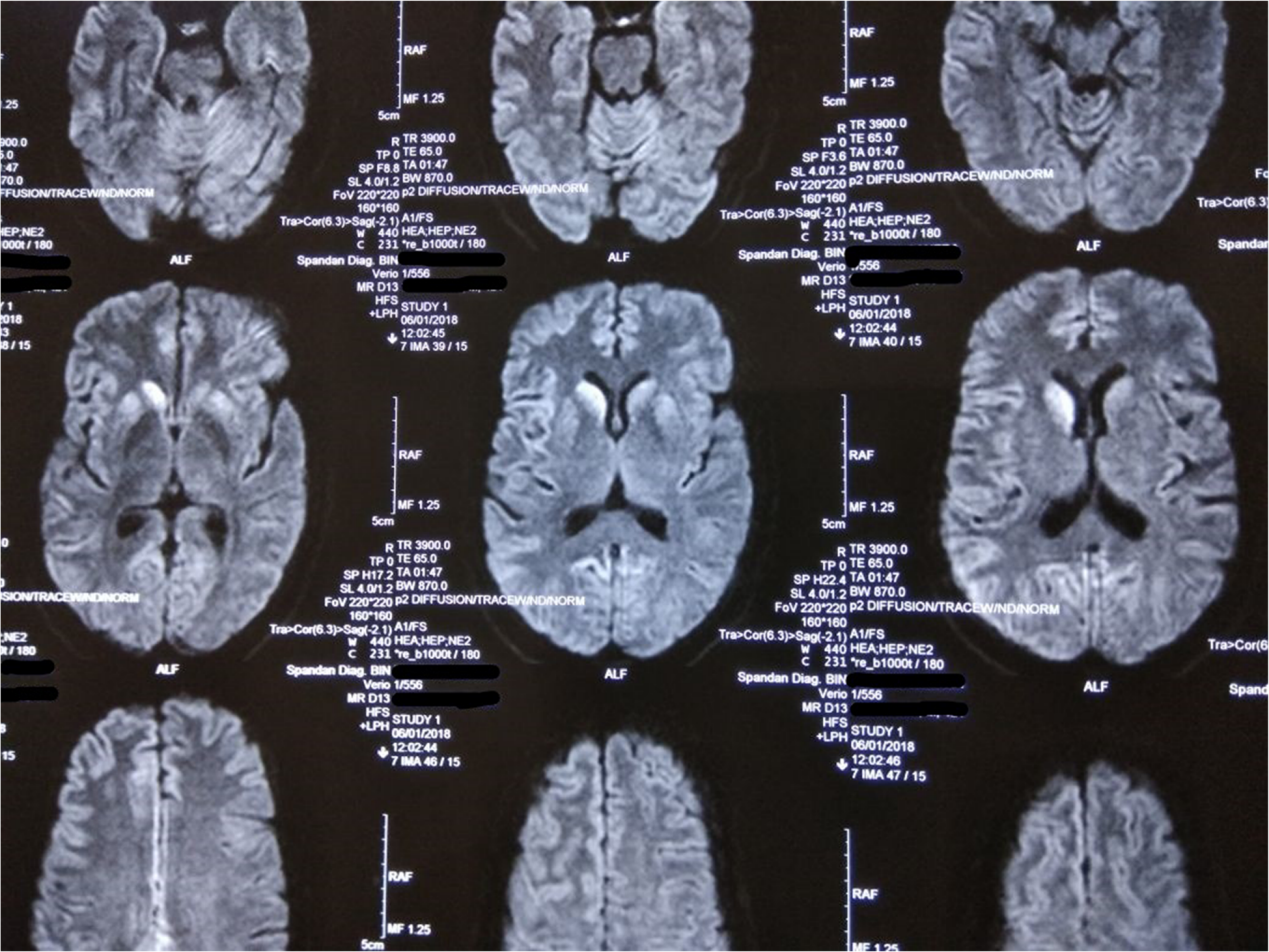
Magnetic resonance imaging brain diffusion-weighted sequence revealed hyperintensity in bilateral caudate nucleus and putamen. Diffusion restriction is also observed in bilateral parieto-occipital and insular cortex (more on right side) resembling typical cortical ribbon pattern of sporadic Creutzfeldt–Jakob disease

**Fig. 2:**
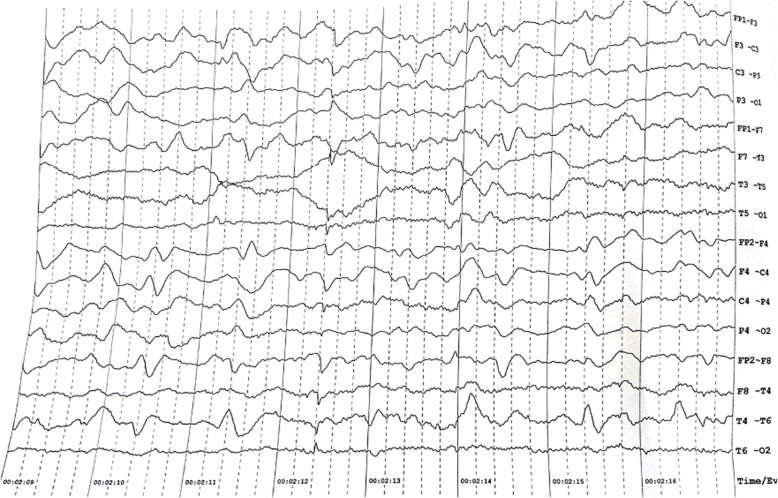
Electroencephalography shows diffuse slowing of background activity accompanied by intermittent slow biphasic and triphasic waves (duration > 200 ms) with suggestion of periodic sharp wave complexes

A 42-year-old woman from India (Asian by ethnicity) without any known comorbidities developed gait and stance unsteadiness around 1 month prior to presenting to us. It was rapidly followed by development of tremulousness in both hands, particularly while reaching for a target. Her first medical contact was through an orthopedic surgeon when she suffered a fall at around 2 weeks after the onset of her illness. At that time, she was diagnosed was having a fracture of her right tibia and received plaster casting of her right leg. However, her neurological illness continued to go downhill as she developed intermittent abnormal twisted posturing of her right hand, suggestive of focal dystonia. Subsequently, she developed progressive deterioration of her cognitive function for around 2 weeks before coming to our care. Reduced attention span, impairment of short-term memory, behavioral abnormality, and language problems in the form of comprehension difficulty as well as irrelevant talking were the major features at the onset of her cognitive disturbance. On detailed questioning, her family members admitted to the presence of intermittent brief rapid involuntary jerks involving one limb at a time, suggestive of myoclonic jerks, which used to persist during sleep as well. Over the course of 3–4 days prior to admission, her higher mental function deteriorated severely enough amounting to akinetic mute state. There was no family history of similar illness. On neurological examination, she was found to have akinetic mute state, paratonia in both upper limbs and left lower limb, intermittent focal myoclonic jerks, and bilateral extensor plantar response. Keeping all these in mind, differential diagnoses were formulated: encephalopathy which might have been due to metabolic factors or an autoimmune process, or an infective pathology such as prion disease. She was investigated thoroughly; her blood count and metabolic parameters were within normal limits. Anti-thyroperoxidase (TPO) antibody in serum turned out to be negative. Anti-nuclear factor was also negative. CSF cytology and biochemical parameters were within normal limits and culture was negative. Tests for syphilis, human immunodeficiency virus (HIV), herpes simplex virus, human herpes virus-6, C-reactive protein, folate, vitamin B_12_, erythrocyte sedimentation rate, and homocysteine levels were all negative. Paraneoplastic markers in CSF also came to be negative. MRI of her brain revealed bilateral caudate nucleus and putaminal hyperintensity in T_2_/fluid-attenuated inversion recovery (FLAIR) sequence as well as in diffusion-weighted imaging (Fig. 1). Restricted diffusion was also observed in multiple cortical areas, mostly in parieto-occipital and insular regions bilaterally (more on right side), resembling ribbon pattern. An EEG showed diffuse slowing of background activity with periodic sharp wave complexes along with intermittent triphasic waves (Fig. 2). A diagnosis was provisionally established based on the positive result of 14-3-3 protein detection in CSF.

Once the diagnosis and prognosis were conveyed to our patient’s relatives, they decided to take her back to a local hospital for terminal care. The details of the follow-up, therefore, could not be obtained in this case.

## Discussion

As we sum up the case, cerebellar ataxia was the dominant feature at onset, which was rapidly followed by focal dystonia. Cognitive decline appeared somewhat late in the temporal course of events accompanied by typical myoclonic jerks. Our patient was in a terminal akinetic mute state within a span of a mere 30 days. Keeping in mind the clinical scenario, short duration (1 month) of illness, and rapid downhill course, our top differentials were primary and secondary brain neoplasms, metabolic derangement, infective encephalopathy, autoimmune (including paraneoplastic) encephalitis, and prion disease. However, all the available metabolic parameters were within normal limits. Infective or autoimmune encephalitis was also discounted after CSF analysis and culture turned out to be negative. Paraneoplastic markers in CSF also were non-contributory. As diffusion-weighted sequence of MRI of our patient’s brain revealed bilateral caudate hyperintensity as well as ribbon pattern in cortex, suggestive of sporadic CJD, we concentrated on prion disease. Serial EEG recordings in our patient revealed gradual slowing of the background activity with more obvious appearance of bi-hemispheric triphasic waves and nearly typical periodic sharp wave complexes. After 14-3-3 protein was detected positive in CSF sample, a diagnosis of probable sporadic CJD was made according to the current European diagnostic criteria (2010) [4].

In young patients with rapidly progressive dementia, when CJD is in the list of differentials, we mostly refer to variant CJD. Now the question remains how variant CJD was ruled out here. The World Health Organization (WHO) listed diagnostic criteria for variant CJD in 2001. In this report, definite variant CJD diagnosis is based on a progressive neuropsychiatric disorder and pathologic confirmation. Variant CJD diagnosis is probable if: (a) the illness lasts longer than 6 months and other typical clinical signs for variant CJD occur; (b) EEG does not show the clinical characteristics of sporadic CJD; and (c) MRI shows bilateral symmetrical pulvinar high signals. The most important segment of the MRI criteria is “pulvinar sign” [5]. It is obvious from the above-mentioned criteria that there was no way to consider variant CJD as a possibility in our patient.

Of late, the occurrence of sporadic CJD is being increasingly recognized in young patients. This particular group of patients with young-onset CJD is somewhat unusual as they share some features with both variant CJD and typical old age sporadic CJD. In a large series of patients with young-onset sporadic CJD, studied by Boesenberg *et al.*, it was observed clearly that cases of sporadic CJD in young patients were not only clinically distinct from older age group cases but also had some significant differences in radiological and electrophysiological aspects [3]. A high prevalence of psychiatric features was noted among these patients. Slower progression of neurological deterioration and a longer disease course, somewhat similar to variant CJD, were also revealed. Typical abnormalities in MRI of the brain were found less frequently in young patients with CJD, whereas suggestive EEG abnormalities were rarely detected in this series. Rather, the diagnosis remained heavily dependent on the result of 14-3-3 in CSF, if other suggestions were available.

Coming to the Indian scenario, a total of 69 cases from different parts of India were recorded in the CJD registry at the National Institute of Mental Health and Neurosciences (NIMHANS), Bangalore, from 1968 to 1997. There were 36 pathologically verified “definitive” cases and 33 “possible” cases of CJD based on clinical features and perhaps EEG. In the article on epidemiology of CJD in India, by Satishchandra and Shankar, the age range was 34 to 76 years among a total of 30 cases [6]. EEG abnormalities were found in a majority of the cases. Interestingly, two cases of hyper-acute clinical presentation were reported in this study [6]. Three other large series of sporadic CJD were available subsequently. Mehndiratta *et al.* in their study of 10 cases found 3 patients below 50 years of age [7]. All three cases presented with abnormal behavior at the onset. The duration of illness in one of them was 3 months and in the other two were 10 months and 14 months. EEG abnormality was noted in all the cases [7]. In the series by Biswas *et al.*, a male patient of 39 years, who was reported to have phenotypic features of probable sporadic CJD, had visual hallucination and rapidly progressive dementia as the initial manifestation [8]. Although disease duration was only of 2 months in this case, he was actually lost in follow-up. Both EEG and MRI abnormalities were noted in this case [8]. In a recent article by Chandra *et al*., 14 cases of probable CJD were observed, among which 8 were in the age group of 60 years or above [9]. Details of the young-onset cases were not separately elaborated in this series [9].

With regard to our case, some unusual points were noted: cerebellar ataxia at the onset, relatively delayed involvement of cognitive domains, much too rapid deterioration with a short duration of illness, typical MRI picture of sporadic CJD, and highly suggestive sequential EEG changes. However, almost no room for doubt was left as 14-3-3 protein was positive in CSF. Hence, we believe that our patient exemplifies a case of young-onset sporadic CJD with some odd phenotypic features.

## Conclusion

Sporadic CJD in young individuals is a rare occurrence. In the existing literature, the described phenotypes of young-onset cases clearly suggest that they are distinct from older-onset CJD from the perspective of brain imaging and EEG, regardless of their apparent similarities in clinical features. However, the case reported here contradicts this notion and possibly represents a different subset of young-onset sporadic CJD. Noteworthy observations in the present case were cerebellar ataxia as the initial symptom, relatively delayed onset of dementia, quick deterioration with a very brief duration of illness, typical MRI findings of sporadic CJD, and highly suggestive sequential EEG changes. Therefore our case not only adds to the tally of cases of young-onset sporadic CJD, but also points to an expanding spectrum of the disease itself.

## Abbreviations

CJD: Creutzfeldt–Jakob disease
CSF: Cerebrospinal fluid
EEG: Electroencephalography
FLAIR: Fluid-attenuated inversion recovery
HIV: Human immunodeficiency virus
MRI: Magnetic resonance imaging
NIMHANS: National Institute of Mental Health and Neurosciences
PrPC: Normal form of prion protein
PrPSc: Abnormal form of prion protein
TPO: Thyroperoxidase
WHO: World Health Organization
